# A Reliable Handoff Mechanism for Mobile Industrial Wireless Sensor Networks

**DOI:** 10.3390/s17081797

**Published:** 2017-08-04

**Authors:** Jian Ma, Dong Yang, Hongke Zhang, Mikael Gidlund

**Affiliations:** 1 School of Electronic and Information Engineering, Beijing Jiaotong University, Beijing 100044, China; 14111026@bjtu.edu.cn (J.M.); hkzhang@bjtu.edu.cn (H.Z.); 2 Department of Information Systems and Technology (IST), Mid Sweden University, Sundsvall 85170, Sweden; mikael.gidlund@miun.se

**Keywords:** Industrial Wireless Sensor Networks (IWSNs), handoff triggering, reliable communication, mobility aware

## Abstract

With the prevalence of low-power wireless devices in industrial applications, concerns about timeliness and reliability are bound to continue despite the best efforts of researchers to design Industrial Wireless Sensor Networks (IWSNs) to improve the performance of monitoring and control systems. As mobile devices have a major role to play in industrial production, IWSNs should support mobility. However, research on mobile IWSNs and practical tests have been limited due to the complicated resource scheduling and rescheduling compared with traditional wireless sensor networks. This paper proposes an effective mechanism to guarantee the performance of handoff, including a mobility-aware scheme, temporary connection and quick registration. The main contribution of this paper is that the proposed mechanism is implemented not only in our testbed but in a real industrial environment. The results indicate that our mechanism not only improves the accuracy of handoff triggering, but also solves the problem of ping-pong effect during handoff. Compared with the WirelessHART standard and the RSSI-based approach, our mechanism facilitates real-time communication while being more reliable, which can help end-to-end packet delivery remain an average of 98.5% in the scenario of mobile IWSNs.

## 1. Introduction

Nowadays, growing demands to improve process efficiencies, comply with environmental regulations, and meet corporate financial objectives faced by a number of companies, which makes the industry marketplace competitive [1]. With a growing number of intelligent devices, mobile devices have a major role to play in industrial production. The roadmap of Factories of the Future indicates that mobility support is one of the main requirements in the wireless scenario [2]. Meanwhile, it is necessary to integrate such characteristics into the key technology of Industry 4.0 [3]. As an emerging class of wireless sensor networks (WSNs), IWSNs are indispensable in industrial production which aim to increase productivity and profit, decrease cost and provide convenience. However, the issued IWSNs standards, e.g., WirelessHART [4] and ISA100.11a [5], introduce mechanisms such as time division multiple access (TDMA) to focus on monitoring for fixed devices. Research on mobility support in IWSNs has been limited because of the specific constraints linked to the specific requirements of the industry. Since the applicability of IWSN protocols is significantly limited to monitoring and control systems by the lack of mobility support [6], this paper presents a new mechanism to deploy a reliable IWSN for applications involved in the mobility of devices, workers or robots.

The common solution for mobility in traditional WSNs is a periodic reception of messages from coordinators or access points to the node. However, this kind of solution works best with the star topology, which lacks scalability, and the contention-based MAC protocol will deteriorate the performance of mobility support. Compared with traditional WSNs, supporting mobility in IWSNs involves more challenges that the adaptation of the complicated resource scheduling needs to be dynamic for the movement [7]. Moreover, there are more interference and unstable factors affecting the performance of wireless communication, such as metallic shelter or electromagnetism. They also affect the accuracy of handoff triggering and easily cause the ping-pong effect while supporting mobility. Generally, the ping-pong effect during handoff and registration is a critical problem for all mobile wireless networks, especially in centralized, TDMA-based IWSNs. Moreover, the effect of harsh and multivariate environments means these challenges are difficult to be solved [8]. For instance, the accuracy of triggering and the time of registration are the two most important aspects of the handoff mechanism. Traditional approaches to the metrics of triggering are based on setting a threshold for radio signal strength indication (RSSI) such as [9,10]. Though the RSSI can evaluate the possible state of the channel [11,12], the large number of lost packets and varied environment are not taken into consideration, which causes false triggering or longer handoff delay [13]. There is also the disputation that RSSI can only indicate some possible anomalous behaviour of devices instead of the packets delivery estimation [14,15]. False triggering not only impacts the timeliness of industrial data but makes the network unstable, which results in a vicious spiral. Such a reaction also increases energy consumption due to unnecessary handoff. However, the problem of frequent registration stems from the fact that the mobile node immediately registers a new link once handoff is triggered. The difference between mobility awareness and the link estimation during network formation is the coherence time for measurements collection. The measurements should be easily obtained and it is necessary to consider the changes of slot scheduling. Generally, both bad channel condition and actual movement may trigger handoff. To find a correct solution to the problem on mobile handoff, the actual movement must be identified. A few solutions were introduced in [16], which can overcome this problem based on the limited communication range of radio and its traditional handoff mechanism.

This project is a collaboration with a company which specializes in industrial machine production. All the welding machines in the production site should be monitored and controlled. Since the parameters, e.g., electric current, voltage, output power and other states, need to be reported to a server in time, the network communication should be reliable and real-time. However, the welding machines always operated to the huge metallic devices by workers and it is not necessary to equip lots of machines due to the cost of installation. Since a welding machine need to weld any part of the device or the other metallic devices at any time, the mobility support in the network communication becomes an urgent requirement. Moreover, the multi-hop wireless sensor network needs to maintain the delivery of monitored data. The inefficient handoff may break the delivery and even increase the transmission delay. The common problem for mobility supporting is the ping-pong effect that a node frequently triggers the handoff by some factors like interference or mobility. Once the node registers a new link, it may trigger the handoff again, which consumes much energy and increases the end-to-end delay. Therefore, this paper proposes an effective handoff mechanism to solve the problem of ping-pong effect during handoff and the registration after handoff. We start by integrating multiple metrics to hold the switching handoff and guarantee the accurate trigger by using the theory of fuzzy logic. Then, to avoid redundant registration, we divide the scenario of handoff into two cases caused by static link failure and actual movement, respectively. In our mechanism, the mobile nodes identify which cases to process by evaluating their mobile state instead of registering a new link immediately. This means that unnecessary handoff and registration are avoided by augmenting the independence of mobile nodes. Another contribution of this paper is that the promotion of packet delivery and the decrease of energy consumption are proved by not only conducting an experiment on our testbed but implementing it in practice.

The rest of this paper is organized as follows: Section 2 presents related work and Section 3 describes the prototype of the system and the network models. The condition of triggering and our proposed mechanism are presented in Section 4 and Section 5 respectively. Section 6 provides the experimental evaluation of both testbed and factory. Finally, the paper is concluded in Section 7.

## 2. Related Work

Solutions such as [17,18,19,20] have been proposed to support mobility in WSNs. Unfortunately, few of these protocols cater to the demand of reliability and timeliness in industry since their contention-based media access control (MAC) protocols cannot provide deterministic packet delivery. Moreover, the harsh and varied environments may deteriorate the stability if applied them into industrial field. Therefore, this paper mainly concentrates on TDMA-based and centralized solutions which can provide the deterministic communication. In some solutions, the transmission scheduling is designed to make up the communication of mobile nodes. In [21], Gonga et al., present a cross layer architecture which combines the MAC and routing layers to achieve energy efficient communication. The costly ability of the multi-channel transceiver is used to allow the cluster-heads to schedule traffic dynamically. A mobility-aware scheduling algorithm is proposed in [22], where the mobile nodes in the algorithm can dynamically associate with the infrastructure nodes by reserving bandwidth for potential communication paths. However, the number of mobile nodes and their moving path are so unpredictable that it cannot be used to design the transmission scheduling. Thus, the complexity of the mobility solution is greatly increased when supporting multi-hop, large, and mesh networks is the basic requirements. As previously mentioned, the accuracy of triggering and the time of registration are two important aspects to consider. For the former aspect, the triggering rule of most solutions is that if the RSSI of the communication link between the mobile node and the current parent is below a predefined threshold then the mobile node will trigger a handoff. However, the suitability of the sole measurement of RSSI in an industrial environment was argued in [23,24]. The theory of fuzzy logic which is used to predict and support mobility in wireless network is not rare either such as [25,26]. In [27], it proposes a reliable handoff procedure, where nodes periodically send a probe message to their access point and expect acknowledgement. The triggering accuracy is improved by using the fuzzy logic rule to combine the velocity and radio signal strength. The problem is that the deterministic delivery of periodic message and acknowledgement cannot be guaranteed. Meanwhile, a fuzzy logic-based mobility controller is designed to describe the degree of triggering in [28] by obtainning the linguistic variables. Many fuzzy rules should be predefined on each node, and more importantly, they do not involve the reliable handoff procedure to avoid the key problem of ping-pong effect. The periodic characteristics is not considered in its controller. As for the time of registration, the state of the mobile node should be evaluated or predicted during its movement. In [29], it uses historical data of radio connectivity between users and static sensor nodes to predict future paths, and another similar solution presents a mobility aware technique of low-latency deterministic network by analyzing and minimizing the dissociation time of the mobile node [30]. Though these solutions can decrease the handoff latency within an over four-hop network, they have not solved the ping-pong effect problem well. In this paper, we employ the ordered weighted average operator to improve the accuracy of handoff triggering, which relates to the theory of fuzzy logic, and more importantly integrate it into the resource scheduling to provide the more reliable communication during handoff. Moreover, the evaluation of actual mobile state is added after triggering handoff to avoid the ping-pong effect and frequent registration.

## 3. System and Network Model

In this section, we will introduce the use case and the whole system model of the proposed mechanism. We denote the integral system developed by our team as the WirelessCAN system.

### 3.1. System

**Figure 1:**
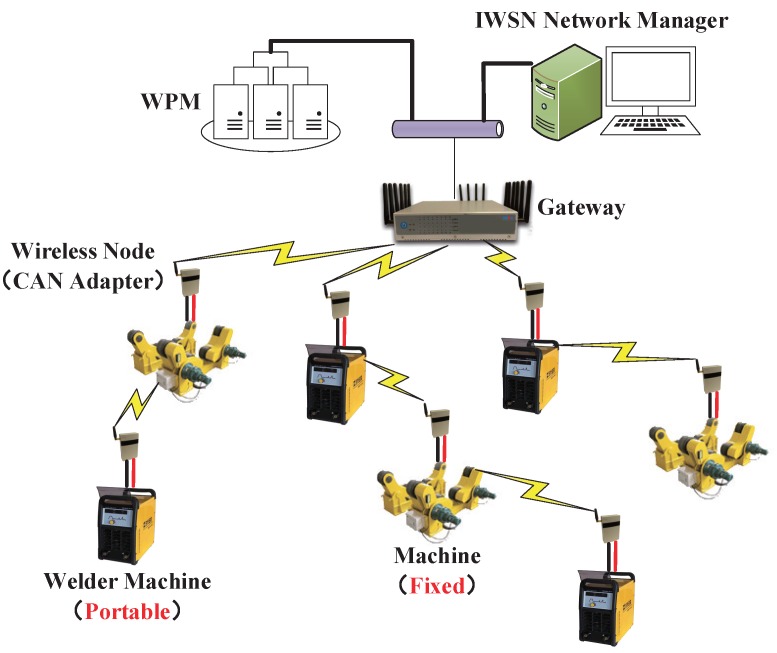
The WirelessCAN system prototype including fixed and portable devices.

The research problem in this paper is a problem encountered in a real industrial application. The system aims to monitor all working parameters of welder machines in production as shown in Figure 1. Therefore, we developed a WirelessCAN system, where the wireless network communication is based on the WirelessHART standard. The communication between welder machines (WMs) and sensor nodes is through the controller area network (CAN) which is the definition of a high performance communication protocol for serial data communication and the data delivery between sensor nodes and the welding parameter manager (WPM) is through a multi-hop wireless sensor network. A description of the main components in WirelessCAN system is presented in the following.

- A W-CAN node is a wireless communication and processing device responsible for the data conversion between the CAN interface and wireless protocol. It can also relay packets to form a multi-hop mesh network.
- A Gateway is an intermediate device able to convert the protocol between wired and wireless. It classifies the data from all W-CAN nodes and sends the data to a server, such as a WPM or Network Manager.
- A Network Manager is a centralized controller of the whole IWSN. All the resources of communication between W-CAN nodes are scheduled in the Network Manager at the network joining process.

A WirelessCAN system has provided reliable monitoring and control. The limitation is that it only provides for the static networks. However, some of the WMs need to be frequently moved to other weld sites in a real production scenario. This is liable to cause packet losses when mobile W-CAN nodes need to rejoin the network. Moreover, if a node is responsible for relaying packets to other nodes, it will result in more packet losses. Therefore, a new mechanism should be designed to solve the problem in a mobile scenario.

### 3.2. Network Model

The network is composed of a collection of infrastructure nodes that form a multi-hop network. In this paper, the Network Manager and the gateway are merged into a centralized controller *g*, which is responsible for network management. The set of nodes is denoted as $V=\{v_{1},v_{2},...\}$, and one of these nodes is in the moving state, we use MN to denote it. Thus, every node can be turned into a MN possibly. Two kinds of flows are defined to support monitoring and control applications. We use $f_{i}^{P}:\langle v,p_{i},\varphi_{i},d_{i}\rangle$ to denote the periodical upstream flow, where *v* is the generator of flow $f_{i}^{P}$ and $\varphi_{i}$ is the phase of dedicated slot allocation in the superframe. Each flow $f_{i}^{P}$ periodically generates a packet at period $p_{i}$ which originates at *v* and has to be delivered to *g* within the deadline $d_{i}$. $f_{i}^{C}:\langle v,d_{i}\rangle$ represents an event-based flow of control applications which routes from *g* to *v*.

**Figure 2:**
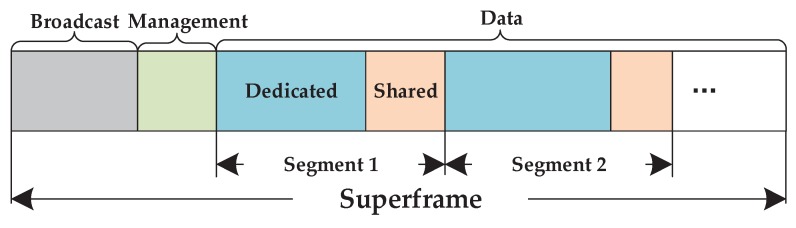
The structure of a superframe.

All transmissions in each flow are assigned 10 ms slots by Network Manager according to WirelessHART, which construct a periodical superframe. The superframe consists of three parts, which are broadcast slots, management slots and data slots as shown in Figure 2. To guarantee the reliable and real-time transmission, the part of data slots scheduling is according to the SSA algorithm in [31], which is divided into the segments by the hop of transmission for all flows. Each segment comprises dedicated slots and shared slots. Two ensuring nodes to do the half-duplex communication occupy the dedicated slot. While the shared slot is reserved for the nodes which need retransmission. Each transmission can be tried for three times at most, including the initial transmission in dedicated slot and two retransmissions in shared slots. Generally, all flows should complete transmission within a superframe, thus the length of superframe *L* depends on the sampling period and the deadline, where it needs to satisfy $d_{i}\leq L\leq p_{i}$ regardless of monitoring and control flow. Spectrum diversity gives the network access to several channels defined in the IEEE 802.15.4 physical layer and allows per-time slot channel hopping. All available channel in channel hopping scheme is evaluated by all the time-slot transmission. Nodes periodically report the performance of each transmission to Network Manager, which will construct a channel blacklist. Therefore, the inspection of available channels is supported by the original WirelessHART standard.

## 4. Trigger Condition

The accuracy of handoff triggering mostly depends on the selection of both metrics and integration operator. This section will introduce the preliminary for the handoff mechanism, including the window of historical information, triggering metrics and integration of metrics.

### 4.1. Window of Historical Information

Nodes proactively realizing the state of mobility is key for advanced handoff mechanisms. To be aware of mobility in time, the number of valid measurements and the time ranges limitation for the valid measurements should be carefully considered. We call the time ranges limitation for the valid measurements as the window of historical information, which is denoted by *W*. A wide window causes slow discovery and deficient statistics give insufficient evidence of the varied state. The difference between mobility awareness and the link estimation during network formation is the coherence time for measurements collection. The link estimation need to decide a more reliable link for each node, which is estimated by a lots of consecutive transmissions. However, the mobility awareness only depends on the periodical data packets to make a fast trigger decision. According to [32], the quality of channel is not constant in time, especially in industrial environments. Thus, the setting of *W* correlates with the speed of node movement and the period of packet transmission.

**Figure 3:**
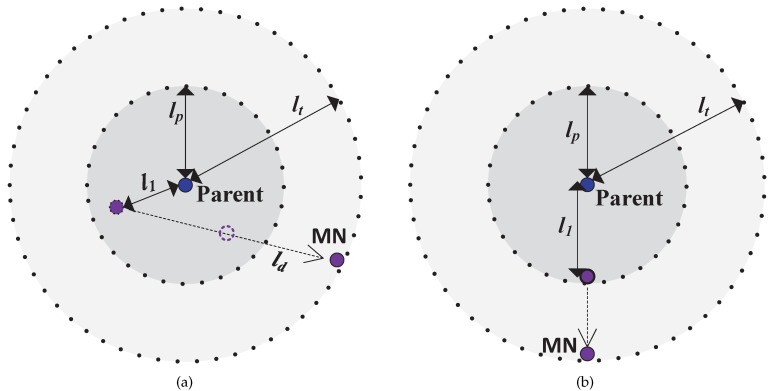
Normal case and worst case where *MN* is moving away from its parent. (**a**) The normal case; (**b**) The worst case.

We assume that the maximum speed of node movement is V and 10×L is the period of packet transmission. In our triggering approach, two types of range are defined to differentiate the quality of communication. We assume that the circles with radius of $l_{p}$ and $l_{t}$ represent two boundaries of the transmission quality during node communication. The circle with $l_{p}$ represents the good-quality boundary and another one represents the limited boundary of the communication quality. Nodes can have high packet delivery with their parent within the good-quality range, but the basic performance of such delivery cannot be guaranteed when the *MN* exceeds the limited boundary. Thus the radius is also expressed as the communication quality though it is not a circle in practice. *MN* needs to realize mobility and trigger the handoff process before exceeding its parent’s limited communication boundary. As shown in Figure 3a, *MN* is currently $l_{1}$ away from its parent, and $l_{d}$ is the dynamic length of *MN*’s movement until it reaches the limited boundary. In Figure 3b, the worst case is that *MN* is very close to the good-quality boundary and it moves away from its parent along the radius. Thus we have $l_{d}\geq l_{t}-l_{1}\geq l_{t}-l_{p}$. We define *t* as the time interval from the beginning of the movement to the handoff triggering. It has to meet $t\leq t_{worst}$. The $t_{worst}$ is the minimum interval to discover the mobile state and is expressed as

(1) $$t_{worst}=\frac{\lambda (l_{t}-l_{p})}{V}$$

where λ is the factor of interference. Therefore, the length of window *W* can be calculated by

(2) $$W=\max\left\{\left\lceil \frac{t_{worst}}{10\times L}\right\rceil ,W_{min}\right\}$$

where ⌈·⌉ represents that the result must be fixed to the given time unit such as superframe in this paper and $W_{min}$ refers to the minimum window able to observe the variation.

### 4.2. Triggering Metrics

The combination of several vital metrics can guarantee more accurate handoff triggering. In this paper, we focus on three aspects for the evaluation of triggering time.

**Metric 1.** Moving State (**MS**)

Moving state is one of the metrics vital for the determination of the handoff triggering. The node extracts the RSSI value in each of the received packets and records them with a current timestamp. Next, the variation rate of the RSSI value is noted to evaluate the general state of movement in all ends of the superframe. In TDMA, we use slot as time unit and we stamp each RSSI value with the absolute slot number (ASN) which denoted by $s_{x}$. Thus a collection of observed data in *W* is $\left\{r\left(s_{0}\right),r\left(s_{1}\right),...,r\left(s_{m}\right)\right\}$, where $r(s_{x})$ is the RSSI value extracted by a packet in the $s_{x}$th slot and *m* is the number measurements. Each node receives at least one packet including acknowledgement within one superframe, thus $(s_{m}-s_{0})/L\geq W$. Finally, the movement is evaluated by means of establishing a unary linear regression model where signal strength and corresponding time construct the linear relationship. We use *k* to denote the variation rate of signal strength, which is expressed as

(3) $$k=\frac{\left|\sum_{i=0}^{m}\left(s_{i}-\bar{s}\right)\left(r\left(s_{i}\right)-\bar{r}\right)\right|}{\sum_{i=0}^{m}{\left(s_{i}-\bar{s}\right)}^{2}}$$

where $\bar{s}$ and $\bar{r}$ refer to their mean values respectively.

**Metric 2.** Channel Condition (**CC**)

Monitoring the condition of the current channel is the second metric to trigger the handoff. Though RSSI is generally able to evaluate the state of movement, it is easily fluctuated, especially in industrial environments because of the effects of reflection, diffraction and absorption produced by metallic obstacles. Therefore, we use signal-to-noise ratio (SNR) to deduce the condition of channel for the potential causal relationship between SNR and transmission performance. The value of SNR can be derived by simply subtracting the noise floor (dBm) from the received signal. The measurement of SNR decreases the effect of noise on late triggering, and it is also easily and freely obtained with every received packet.

**Metric 3.** Packet Delivery (**PD**)

The lower bound of the current link quality is that the packet delivery reaches unacceptable level. The above two metrics are evaluated to predict at what time handoff is triggered. However, handoff needs to be triggered immediately when the packet delivery of the current link is below a certain level. Therefore, the required number of packet transmissions (RNP) is a good choice for the evaluation of packet delivery, which is estimated in transmitter. It computes the RNP by counting all transmissions and retransmissions of each transmitted packet over *W*. Thus, RNP is calculated as

(4) $$\mathrm{RNP}=\frac{N_{t}}{N_{s}}$$

where $N_{t}$ is the total number of transmissions and retransmissions and $N_{s}$ is the number of successfully transmitted packets. Based on TDMA protocol, the receiver should acknowledge each data packet transmitted by transmitter. These replied acknowledgements are counted as $N_{s}$.

### 4.3. Integration of Metrics

As discussed before, none of these metrics like RSSI or SNR could make an accurate decision to trigger handoff on their own due to the varied and harsh environments. However, Fuzzy Logic theory is an approach where integrating three metrics. As the decision-making schemes is developing on the electronic area, Fuzzy Logic theory can be used to obtain the more accurate decision based on linguistic variables and inference rules. The gap to combine multiple conflicting or independent objectives by classical logic can be bridged. The degree can be replaced with a linguistic variable whose values are words or sentences in natural or artificial language. Formally, a fuzzy set *A* in X={x} is characterized by its membership function $\mu_{A}:X\to \left[0,1\right]$ and $\mu_{A}\left(x\right)$ is interpreted as the degree, where *x* is the membership of element. It is clear that when *A* is completely determined by the set of tuple $A=\{\left(x,\mu_{A}\left(x\right)\right)|x\in X\}$. If $\mu_{A}\left(x\right)=1$ or 0, then the fuzzy set *A* becomes an ordinary set. Each metric provides partial information and finally the trigger is determined by predefined fuzzy rules. However, the rules are extreme for practical multiple objective problems. Therefore, extensive studies have been carried out regarding the solution of multiple objective problems by using compensatory operators such as [33,34]. The Ordered Weighted Averaging (OWA) operator introduced by [35] is one of the operators in our mechanism which allows easy adjustment of the degree. Accordingly, “andlike” and “orlike” operators with *n* fuzzy sets are given by

(5) $$\mu_{and}=\beta \min_{i}\left(\mu_{i}\left(x\right)\right)+\frac{1-\beta }{n}\sum_{i=1}^{n}\mu_{i}\left(x\right)$$

(6) $$\mu_{or}=\beta \max_{i}\left(\mu_{i}\left(x\right)\right)+\frac{1-\beta }{n}\sum_{i=1}^{n}\mu_{i}\left(x\right)$$

where β∈[0,1] is a compensatory coefficient. It is recommended in the range [0.5...0.8] according to [36]. Since data has strict requirements in industrial production, the “andlike” operator is more suitable for the result in [37]. Therefore, the calculation of the trigger degree in the following handoff process results from (5). 

## 5. Effective Handoff Mechanism

To improve the accuracy of the handoff trigger and decrease link loss during movement, three steps are executed in our mechanism. In this section, we present the details of the scheme of mobility awareness, handoff and registration. We assume that each node generates a data packet and sends it to network manager at every superframe for monitoring application. For downstream, the Network Manager instructs every node when it is necessary.

### 5.1. Mobility Awareness

**Figure 4:**
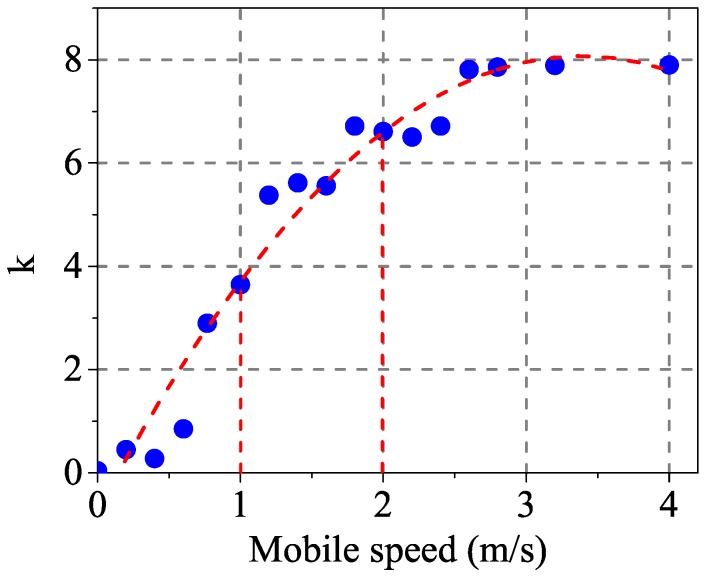
Relationship between *k* and mobile speed.

**Figure 5:**
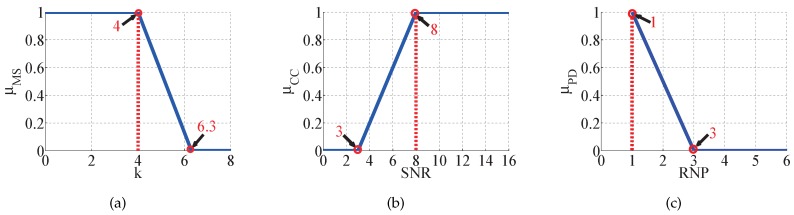
Definition of membership functions: (**a**) is the member function of MS, (**b**) is the member function of CC and (**c**) is the member function of PD.

Each node selects a parent node with a good connection to form a multi-hop spanning tree topology. After joining the network, each node collects metrical information from each transmission and reception. Being an initiative aware mechanism, the node can extract metrics from the acknowledgement of transmission and the reception of command. In addition, each node broadcasts for network management at every superframe. At the end of the superframe, nodes compute the degree of triggering according to the collected metrics within the time window calculated by (2), which is denoted by $\mu (Tr_{W})$. To obtain an accurate trigger decision from the OWA operator, the definition of the membership function is required to map the trigger degree. Moving State, Channel Condition and Packet Delivery are converted to a corresponding degree by defining the values respectively. The respective member functions of three metrics are the linear relationship between the modified measurements and the corresponding degree of metric. To describe the relationship clearly, the importance is to define the key degree of the measurements. These key values of measurements originate from the experimental data and the existing results. Thus, the diagram of the member function can intuitively perform their relationship. For MS, tests are carried out to describe the relationship between *k* and mobile speed as shown in Figure 4. Since the movement of welder machine is generally caused by a worker who need to change the current position. Therefore, we select two values of *k* corresponding to 1 m/s and 2 m/s mobile speed as the key values in membership function $\mu_{MS}$, which is shown in Figure 5a. As for **CC**, there are a number of studies providing a relationship curve between bit error rate and SNR. The membership function of $\mu_{CC}$ is described in Figure 5b, where 3 and 8 are selected as the boundaries of the good channel and bad channel respectively. As a transmitter-side measurement, **PD** is evaluated by counting the number of transmissions and retransmissions and its membership function $\mu_{PD}$ is shown in Figure 5c. Based on the representative standard WirelessHART, the maximum retransmissions is three. Thus it is considered the bound of a bad link when RNP reaches three. RNP = 1 is the bound of the good link where packets are all received successfully.

The linear relationship between collected information and corresponding degree is enough to represent individual metrics. Moreover, it also decreases the complexity of computing and energy consumption. Finally, after the conversion between metrical information and degree, we can obtain the current degree of triggering handoff by Equation (7) according to Equation (5).

(7) $$\begin{array}{cc} \mu (Tr_{W}) & =\beta \cdot min(\mu_{MS}\left(Tr_{W}\right),\mu_{CC}\left(Tr_{W}\right),\mu_{PD}\left(Tr_{W}\right))\\ & +\frac{1-\beta }{3}\left(\mu_{MS}\left(Tr_{W}\right)+\mu_{CC}\left(Tr_{W}\right)+\mu_{PD}\left(Tr_{W}\right)\right) \end{array}$$

Obviously, once such degree is less than the given threshold $\mu_{T}$, the node is aware of its movement and begins the handoff procedure. To provide a more comprehensive assessment, the result is changed to the range in [0...100] in accordance with

(8) $$\mu =100\cdot \mu (Tr_{W})$$

### 5.2. Handoff

**Figure 6:**
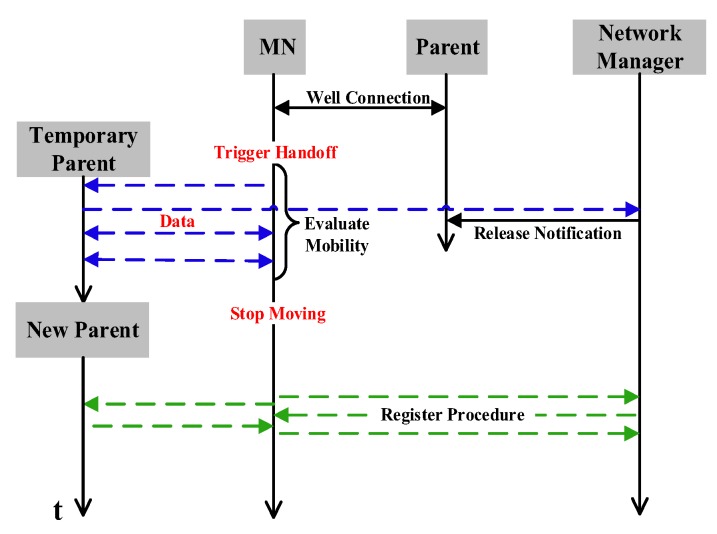
The sequence of handoff procedure.

**Figure 7:**
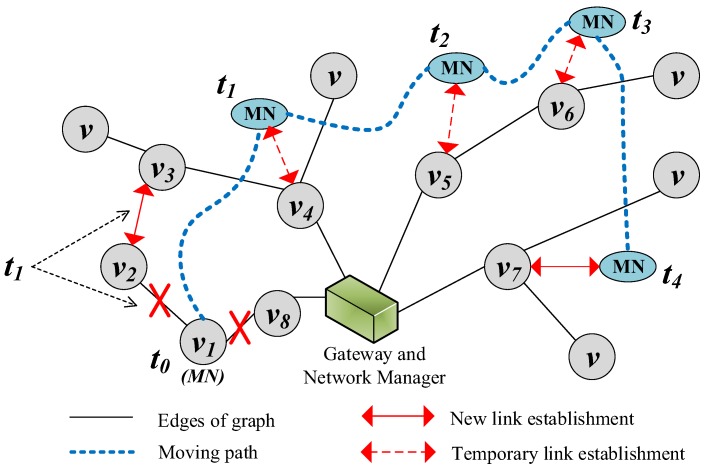
Movement of the mobile node from time $t_{0}$ to $t_{4}$.

The sequence of handoff procedure is shown in Figure 6. After handoff triggering, the *MN* should not leave the network or register a new path immediately, since ping-ping effect during handoff can cost extra communication resource and delivery delay. Therefore, an important feature of our proposed mechanism is that *MN* continuously evaluating its mobile state before registering a new path. The evaluations aim to distinguish between the handoff triggered by interference and real movement. Once *MN* realizes that its state is close to static, it begins a new registration. While on the move, it makes an evaluation every superframe. In Figure 7, we show the procedure of handoff which is designed to avoid the problem of ping-pong effect. At time $t_{0}$, $v_{1}$ becomes *MN* and begins to move along the moving path until time $t_{4}$.

As soon as *MN* triggers handoff procedure, it opens the scan mode from $t_{0}$. To save energy, *MN* only listens to the broadcast nearby, instead of idle listening, since it is still synchronized with the network. We append additional information to the broadcast of each node such as $\varphi_{i}$. Thus, *MN* has a neighbor table $N=\{n_{1},\cdots ,n_{j},\cdots ,n_{m}\}$ during scan mode. Each neighbor can be represented as $n_{j}:\langle v_{i},\varphi_{i},r_{i}^{c},r_{i}^{p}\rangle$, where $r_{i}^{c}$ and $r_{i}^{p}$ are the RSSI values of $v_{i}$’s broadcast measured in the current and previous supreframe respectively. The moving state is evaluated by the following equation.

(9) $$R=\frac{1}{m}\sum_{i}\left|r_{i}^{c}-r_{i}^{p}\right|$$

*R* is the mean of all neighbours’ RSSI variation and *m* is the number of neighbours detected during the last two superframes. When *R* is below the threshold $R_{T}$, *MN* immediately begins to register the new path. Just as $v_{2}$ realizes its link $v_{2}⟶v_{1}$ has broken up at $t_{1}$, it can immediately request for a new link from the Network Manager.

**Figure 8:**
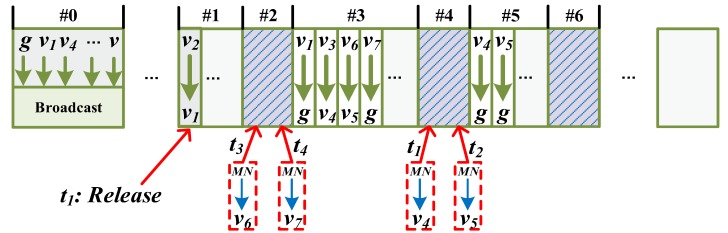
The structure of the superframe and the adjustment when *MN* moves from time $t_{0}$ to $t_{4}$.

Another innovative feature is that *MN* establishes temporary links for transmissions if $f_{MN}^{P}$ and $f_{MN}^{C}$ are generated during the movement. A node is still sampling the data from the welding machine while it is moved to another place. The measurements of moving state evaluation also can be utilized for selecting one of the temporary links. It is different from the WirelessHART standard that the resource of communication will be released by the Network Manager when it is beyond the communication range of its parent. Therefore, the temporary link can improve the delivery, which requires the reliability and timeliness in industry. The segments slot allocation proposed in [31] is scheduled, which makes full use of the sequence of segment and shared slots in this paper. During $t_{0}$ to $t_{4}$, see Figure 7, we assume that $f_{MN}^{P}$ will be generated. While evaluating the moving state, *MN* selects a better infrastructure node to be its parent by comparing the signal strength and the broadcasted parameters. $v_{4}$, $v_{5}$, $v_{6}$ and $v_{7}$ are selected to be parents around time $t_{1}$, $t_{2}$, $t_{3}$ and $t_{4}$ respectively. The scheduling of the superframe is displayed in Figure 8, which is divided into segments. According to $\varphi_{i}$, broadcasted by the selected parent, the temporary link is allocated in the shared slot which belongs to the previous segment $\varphi_{i}-1$. For example, $\varphi_{4}$ and $\varphi_{5}$ are both allocated to #5 at the beginning. Thus, the temporary link is assigned in #4 when $v_{4}$ and $v_{5}$ are selected by *MN* around $t_{1}$ and $t_{2}$ respectively. In the same way, transmissions of $MN⟶v_{6}$ and $MN⟶v_{7}$ are allocated in #2 around $t_{3}$ and $t_{4}$ respectively. Packets of *MN* are aggregated to *g* by the selected parent if there is an aggregation function, or if they are transmitted in subsequent segments of shared slots. As for the downstream flow $f_{MN}^{C}$, it is only generated when the Network Manager indicates the command to the node. We add the address of the selected parent into the packet’s network header, where it helps the Network Manager to decide the real downstream path of *MN*.

### 5.3. Registration

To guarantee network stability, the scheduled superframe and registration need to be adapted for mobility. The infrastructure nodes connected to *MN* should also detect the broken link when *MN* moves to another location. As *MN*’s leaf nodes, they re-register the new links and resources same as the principle of handoff triggering and registration. When it comes to the parent node of *MN*, once it detects that the link to *MN* has broken, it reports to the manager and releases such link resources. For instance, when *g* receives the upstream packets from *MN*, it checks the parent address added by *MN* in the network header. If it is different from the registered parent address, *g* will record it temporarily for the downstream flow. Moreover, the allocated slot resource should be released, thus *g* issues a notification to *MN*’s original parent. Just as the *MN*’s packet arrives through $v_{4}$ at $t_{1}$, *g* sends a notification to $v_{8}$ to release the relative resources related to links $MN⟶v_{8}$ and $v_{8}⟶g$.

## 6. Evaluation and Experiments

**Figure 9:**
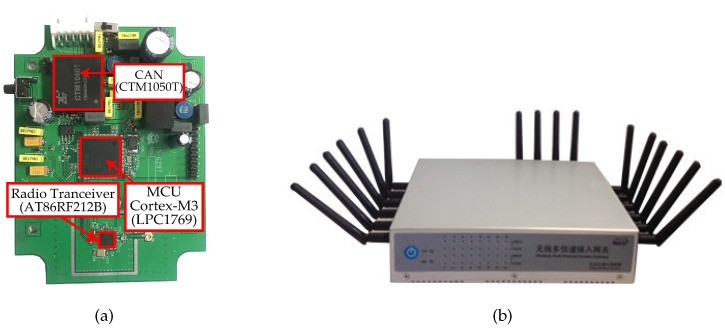
Hardware of WirelessCAN node and Gateway. (**a**) A node; (**b**) A Gateway.

Experiments are carried out not only to decide the related threshold but to evaluate the performance of our mechanism. We implement them in two environments: a testbed and a factory. Our test bed consists of 20 nodes with LPC1769 mote and AT86RF212B radio transceiver as shown in Figure 9a. The power of each node is supplied by two three-voltage batteries. All nodes are centrally scheduled by the gateway displayed in Figure 9b. We integrate the gateway with the Network Manager for convenience. The proposed mechanism works in the MAC layer mostly, including the record of measurement and the calculation of triggering degree. The member function and the corresponding calculation are predefined as a program in all nodes. To facilitate the computation, each nodes only record ten of the best neighbors which have the higher signal strength. An experimental description can be found in the following section and the details of the remaining parameters are listed in Table 1.

The decision of whether or not to handoff is based on locally available information that node collects at a specific time. Thus the performance of the proposed mechanism is assessed by packet delivery. The number of lost packets and expired packets are counted in the gateway. The lost packets are not received by gateway since they exceed the Max Frame Retries. The arrival time of some packets exceeds the deadline, meaning these packets are expired. To increase the packet delivery, the statistics of *MN* is separated from the whole network. Thus, RLP-*V*, RLP-*MN*, REP-*V* and REP-*MN* are used to denote the ratio of the network’s lost packets, *MN*’s lost packets, the network’s expired packets and *MN*’s expired packets, respectively.

### 6.1. Evaluation of Related Parameters

**Figure 10:**
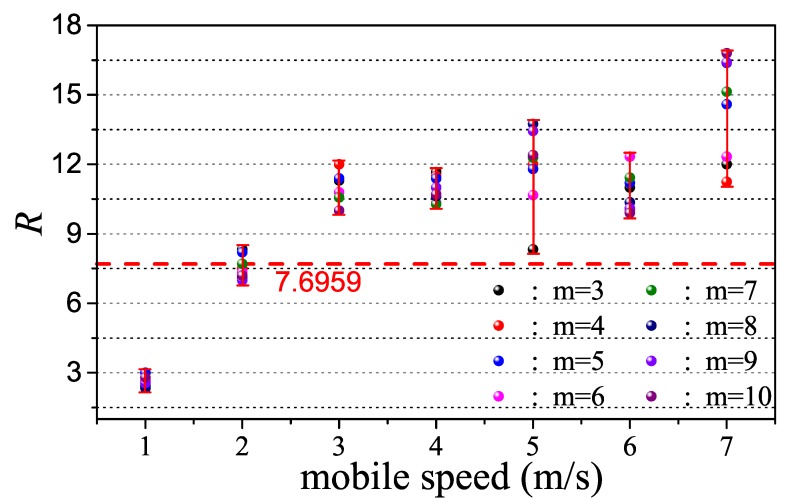
Relationship between *R* and mobile speed.

Before implementing the proposed mechanism, some related parameters should be determined, such as the threshold of the moving state evaluation $R_{T}$ and the threshold of handoff triggering degree $\mu_{T}$. During movement, *MN* determines *R* at each end of the superframe by monitoring infrastructure nodes nearby. The number of monitored nodes is uncertain in practice, which is why we carry out several tests to describe the relationship between mobile speed and the value of *R*. We set a different number of nodes around *MN* for each mobile speed and calculate the corresponding *R*, the results of which are shown in Figure 10. The result with the same mobile speed and different number of neighbor nodes is represented by different colors. To decide the threshold of *R*, we should begin by ensuring a speed value as the boundary. In this paper, we consider a node as moving when its speed exceeds 2 m/s since the movement of welding machine is caused by the workers who need to change their work position. Therefore, we select the average value of *R* in 2-m/s mobile speed as the $R_{T}$, which is 7.7, and such threshold is denoted by the red dash line.

**Figure 11:**
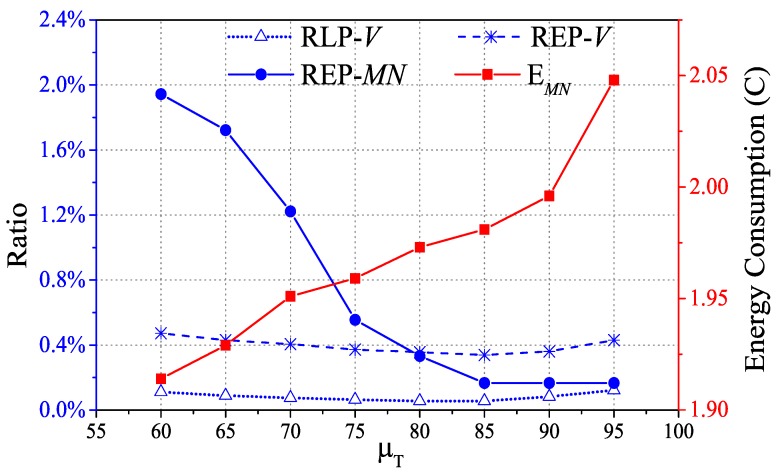
RLP-*V*, REP-*V*, REP-*MN* and energy consumption of mobile node with variation of $\mu_{T}$.

The handoff is triggered by comparing the output of mobility awareness and the predefined threshold $\mu_{T}$. The window of measurement is set to 5 since λ=0.8 is used to convert $l_{t}$ and $l_{p}$ to the performance of packet delivery. The maximum speed supported in this paper is 5 m/s. Different handoff trigger thresholds have influence on not only packet delivery but also energy consumption. Therefore, we conduct the experiments to obtain RLP-*V*, REP-*V*, REP-*MN* and energy consumption of *MN* with eight different $\mu_{T}$ to decide the preferred threshold. The experiments are implemented in our testbed and the environment is described in the following section. The results in Figure 11 show that RLP-*V*, REP-*V* and REP-*MN* are all decreasing until $\mu_{T}$ reaches 85. As $\mu_{T}$ moves up from 85, the value of REP-*MN* tends to be moderate, however, both RLP-*V* and REP-*V* have a growing tendency. Since the larger the $\mu_{T}$ is, the more frequent the handoff, which has a great effect on network topology. However, the performance of the packet delivery is compensated by energy consumption. As the $\mu_{T}$ increases, the *MN* consumes more energy. It is better to select the lower value as $\mu_{T}$ while the network reliability is also guaranteed. Thus, we select 85 as the threshold of triggering degree.

### 6.2. Testbed Experiments

Our testbed positions 20 nodes in a 50 × 50 m${}^{2}$ square at random. Nodes are not too close to each other for practical reasons, and there is no case where *MN* cannot detect any of the infrastructure nodes. The model of mobility is random waypoint [38], it imitates a man catching a node. Each round of the experiment lasts for 30 min. It contains several pause and movement, which sum 10 and 20 min respectively. The pause time is randomly selected within one minute. Considering the length of the superframe, less than four nodes are served by the same parent. Another assumption is that every node takes probability to be a temporary parent of *MN*. The mobility imitates a person walking or running, which is why the speed and direction of movement are not precisely controlled but rather practical. Interference is added by putting Wi-Fi devices around the testbed. The interference is determined by adjusting the Wi-Fi, including frequency, signal strength and quantity. Two levels of interference with packet error rate 2% and 6% are used in the experiments to demonstrate a normal and a bad wireless channel respectively. To facilitate counting the packet delivery and to measure the energy consumption of *MN*, two nodes are considered to be the mobile nodes. The results of *MN* shown in the figures are the average of two *MN*s.

**Figure 12:**
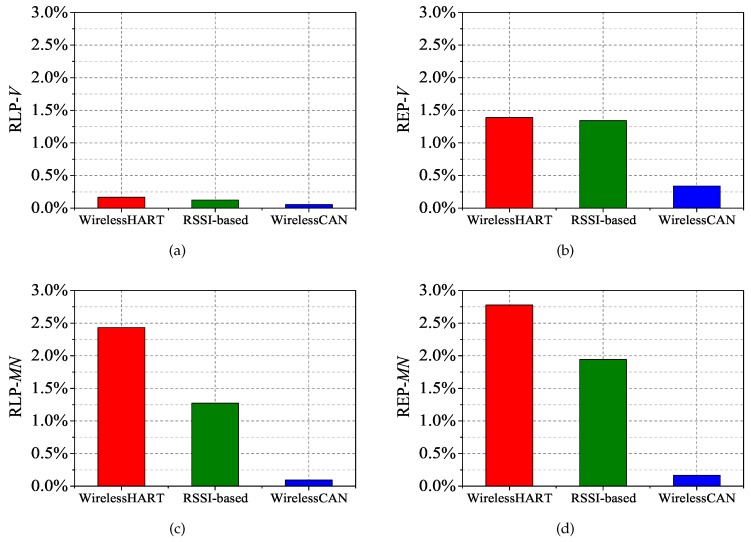
Comparison of RLP-*V*, REP-*V*, RLP-*MN* and REP-*MN* with a 2% packet error rate. (**a**) RLP-*V*; (**b**) REP-*V*; (**c**) RLP-*MN*; (**d**) REP-*MN*.

To evaluate the performance of our proposed mechanism, comparisons of RLP-*V*, REP-*V*, RLP-*MN* and REP-*MN* are made using WirelessHART and RSSI-based handoff. The results of the packet delivery with a 2% packet error rate are shown in Figure 12a–d. Although the RLP-*V* of the two solutions are not much lower than our mechanism, three other metrics show a big gap between WirelessCAN and two other solutions. Firstly, it roughly indicates that the mobility has a great effect on IWSNs without mobility support. Secondly, the common solution of RSSI-based handoff cannot solve this problem particularly well. Compared with WirelessHART, our mechanism decreases 2.34% RLP-*MN* and 2.61% REP-*MN*, even though there are two mobile nodes and the results represent their average value. Meanwhile, the decreases of 1.16% RLP-*MN* and 0.83% REP-*MN* are only contributed by the solution of RSSI-based handoff. Most expired packets in WirelessHART and RSSI-based solutions are caused by the packets of *MN* not being transmitted timely during movement. The reason why the RSSI-based solution causes 1.34% REP-*V* is that the ping-pong effect can occur on any of the nodes due to imprecise triggering.

**Figure 13:**
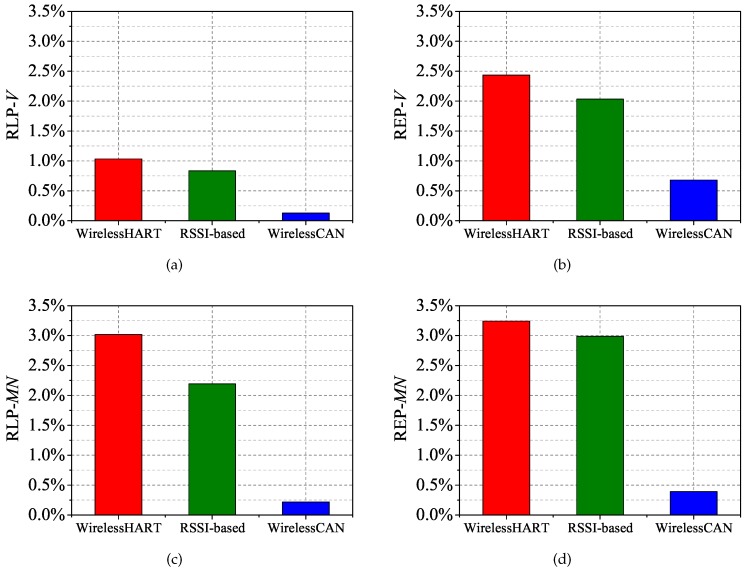
Comparison of RLP-*V*, REP-*V*, RLP-*MN* and REP-*MN* with a 6% packet error rate. (**a**) RLP-*V*; (**b**) REP-*V*; (**c**) RLP-*MN*; (**d**) REP-*MN*.

**Figure 14:**
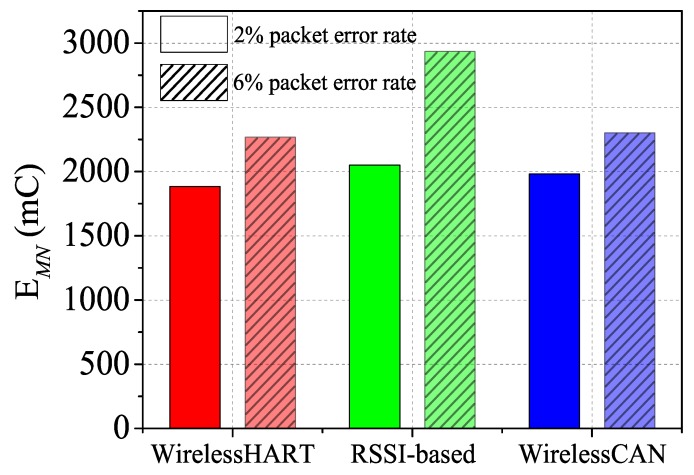
Energy consumption comparison ($E_{MN}$).

As the interference deteriorates to 6% packet error rate, the performance of the WirelessHART and RSSI-based solution is worse still that the performance under 2% packet error rate as shown in Figure 13a–d. The packet error rate is approximately 6%, or even higher in most industrial environments. However, RLP-*V*, REP-*V*, RLP-*MN* and REP-*MN* just respectively decrease 0.07%, 0.34%, 0.12% and 0.22% using WirelessCAN system with interference of 6% packet error rate. The results of comparison between the two levels of interference show that our mechanism is largely unaffected by increasing the interference. However, the more interferences there are, the more instability is generated during triggering when using RSSI-based handoff. Moreover, the ping-pong effect problem in the RSSI-based solution causes more energy consumption. Figure 14 shows the energy consumption of *MN* in each solution, denoted as $E_{MN}$. As demonstrated, $E_{MN}$ of our mechanism is close to WirelessHART, but much lower than the RSSI-based solution. Consecutive registration in WirelessCAN does not increase energy consumption, but in the remaining two solutions it does. The extra $E_{MN}$ in our solution is consumed for the transmission during the movement. Thus our WirelessCAN system improves the reliability under frequent mobility in IWSNs.

### 6.3. Factory Experiments

**Figure 15:**
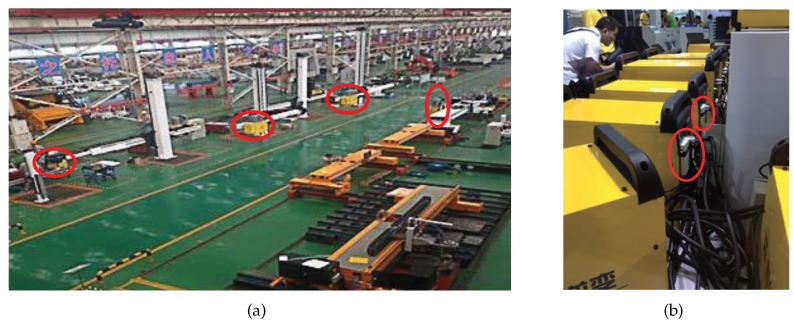
(**a**) The site of implementation and (**b**) the welder machine with the W-CAN node.

As the testbed experiments only evaluate a part of the design in terms of environment and corner cases, we also conduct experiments in a factory. The factory environment is harsher and more multivariate than the testbed. It can have a great effect on the results of experiments, which may be below the current results in the testbed experiments. This makes the practical implementation necessary in order to prove the performance of the proposed mechanism. As shown in Figure 15a, several WMs are marked by red circles on the pushcart and they are all portable. Figure 15b is the welding machine and the W-CAN node.

**Figure 16:**
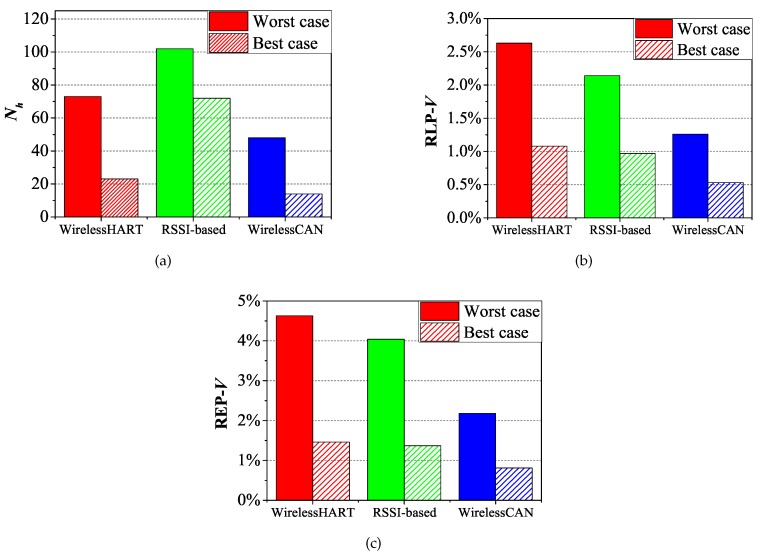
Comparison of RLP-*V*, REP-*V* and the number of handoff in practical implementation. (**a**) The number of handoff; (**b**) RLP-*V*; (**c**) REP-*V*.

Both upstream and downstream flows are supported in the WirelessCAN system to monitor and control all WMs. The practical implementation consists of 12 WMs with W-CAN nodes, all of which could be turned into *MN*s. Thus the metrics of RLP-*MN*, REP-*MN* and $E_{MN}$ are difficult to obtain. However, we obtain the number of handoffs ($N_{h}$) in each solution by counting the number of parent changes in the gateway. On each weekday, five experiments are implemented to amply demonstrate the performance of our mechanism. Table 2 lists the results of the experiments, and Figure 16a–c show the difference of RLP-*V* and REP-*V* between the three solutions by selecting the worst and best results of the five experiments. A varied number of handoffs greatly influence the performance of WirelessHART and RSSI-based solutions. Moreover, a lower number of handoffs not only make the network more stable but save management slots. The results also roughly indicate that the expired and lost packets are reduced when using the WirelessCAN system. On the contrary, other two solutions cannot solve the mobility problem well as the environments become harsher and multivariate. In general, WirelessCAN system can offer reliable packet end-to-end delivery to an average of 98.5% in an industrial factory.

## 7. Conclusions

In this paper, we proposed an mechanism to support mobile devices in industrial monitoring and control systems. Our mechanism improves reliability by concentrating on the accuracy of handoff triggering and the time of registration. We used the theory of Ordered Weighted Averaging operator to improve the accuracy of the triggering decision. We also showed the importance of mobile state evaluation during node movement and introduced a scheme where nodes can complete transmission during movement. Finally, the performance of the proposed mechanism is evaluated and implemented in both a testbed and a industrial factory. The results of testbed experiments showed that our solution respectively reduces 2.61–2.85% and 1.78–2.6% expired packets of mobile node compared to the WirelessHART and RSSI-based solution. The WirelessCAN system can also offer a reliable packet end-to-end delivery to an average of 98.5% in an industrial factory.

## Figures and Tables

**Table 1:** Parameter Details.

Parameters	Value
Microcontroller	Cortex-M3
Power of Transmission	3 dBm
Rate of Transmission	250 kb/s
Length of Superframe	1 s
Size of Packet	40 Bytes
Max. Frame Retries	3
Max. Speed of MN	5 m/s
*W*	5

**Table 2:** Results of Practical Implementation.

No.	WirelessHART	RSSI-Based	WirelessCAN
1	(73, 2.63%, 4.63%)	(102, 2.14%, 4.04%)	(48, 1.26%, 2.18%)
2	(64, 1.57%, 3.82%)	(93, 1.29%, 3.28%)	(37, 1.05%, 1.76%)
3	(23, 1.08%, 1.46%)	(72, 0.97%, 1.37%)	(14, 0.53%, 0.81%)
4	(52, 1.44%, 3.52%)	(84, 1.27%, 3.46%)	(39, 0.95%, 1.34%)
5	(32, 0.73%, 1.78%)	(67, 0.69%, 1.81%)	(32, 0.46%, 0.77%)

**Note:** The values in the table represent ($N_{h}$, RLP-*V*, REP-*V*).

## References

[1] Lu C., Saifullah A., Li B., Sha M. (2016). Real-Time Wireless Sensor-Actuator Networks for Industrial Cyber-Physical Systems. Proc. IEEE.

[2] European Factories of the Future Research Association (2015). Factories of the Future. Multi-Annual Roadmap for the Contractual PPP under Horizon 2020.

[3] Li X., Li D., Wan J., Vasilakos A.V., Lai C.F., Wang S. (2017). A review of industrial wireless networks in the context of Industry 4.0. Wirel. Netw..

[4] WirelessHART Specification IEC Standard. http://www.hartcomm2.org.

[5] The International Society of Automation (2009). ISA100.11a-2009 Wireless systems for industrial automation: Process control and related applications. Standard, Sept.

[6] Papadopoulos G.Z., Kritsis K., Gallais A., Chatzimisios P. (2015). Performance evaluation methods in ad hoc and wireless sensor networks: A literature study. IEEE Commun. Mag..

[7] Kumar Somappa A.A., Ovsthus K., Kristensen L.M. (2014). An Industrial Perspective on Wireless Sensor Networks—A Survey of Requirements, Protocols, and Challenges. IEEE Commun. Surv. Tutor..

[8] Al-Nidawi Y., Yahya H., Kemp A.H. Impact of mobility on the IoT MAC infrastructure: IEEE 802.15.4e TSCH and LLDN platform. Proceedings of the IEEE World Forum on Internet of Things.

[9] Caldeira J.M.L.P., Rodrigues J.J.P.C., Lorenz P. (2015). MAC layer handover mechanism for continuous communication support in healthcare mobile wireless sensor networks. Telecommun. Syst..

[10] Lei T., Wen X., Lu Z., Jing W., Zhang B., Cao G. Handoff Management Scheme Based on Frame Loss Rate and RSSI Prediction for IEEE 802.11 Networks. Proceedings of the 2016 International Symposium on Wireless Communication Systems (ISWCS).

[11] Barac F., Gidlund M., Zhang T. (2015). Ubiquitous, Yet Deceptive: Hardware-Based Channel Metrics on Interfered WSN Links. IEEE Trans. Veh. Technol..

[12] Barac F., Caiola S., Gidlund M., Sisinni E. (2014). Channel Diagnostics for Wireless Sensor Networks in Harsh Industrial Environments. IEEE Sens. J..

[13] Cardone G., Corradi A., Foschini L. Reliable communication for mobile MANET-WSN scenarios. Proceedings of the IEEE Symposium on Computers and Communications, ISCC 2011.

[14] Papadopoulos G.Z., Gallais A., Schreiner G. Importance of Repeatable Setups for Reproducible Experimental Results in IoT. Proceedings of the ACM Symposium on PERFORMANCE Evaluation of Wireless Ad Hoc, Sensor & Ubiquitous Networks.

[15] Papadopoulos G.Z., Gallais A., Schreiner G., Jou E., Noel T. (2017). Thorough IoT testbed characterization: From proof-of-concept to repeatable experimentations. Comput. Netw..

[16] Dong Q., Dargie W. (2013). A Survey on Mobility and Mobility-Aware MAC Protocols in Wireless Sensor Networks. IEEE Commun. Surv. Tutor..

[17] Fotouhi H., Alves M., Zuniga Zamalloa M., Koubaa A. (2014). Reliable and Fast Hand-Offs in Low-Power Wireless Networks. IEEE Trans. Mob. Comput..

[18] Dargie W., Wen J. A seamless handover for WSN using LMS filter. Proceedings of the The IEEE Conference on Local Computer Networks.

[19] Papadopoulos G.Z., Kotsiou V., Gallais A., Chatzimisios P., Noel T. (2015). Wireless Medium Access Control under Mobility and Bursty Traffic Assumptions in WSNs. Mob. Netw. Appl..

[20] Peng F., Cui M. (2015). An energy-efficient mobility-supporting MAC protocol in wireless sensor networks. J. Commun. Netw..

[21] Gonga A., Landsiedel O., Johansson M. MobiSense: Power-efficient micro-mobility in wireless sensor networks. Proceedings of the International Conference on Distributed Computing in Sensor Systems and Workshops.

[22] Dezfouli B., Radi M., Chipara O. Real-Time Communication in Low-Power Mobile Wireless Networks. Proceedings of the IEEE Consumer Communications & NETWORKING Conference.

[23] Srinivasan K., Levis P. RSSI is under appreciated. Proceedings of the The Workshop on Embedded Networked Sensors.

[24] Zinonos Z., Vassiliou V. Handoff Algorithms for Industrial Mobile Wireless Sensor Networks. Proceedings of the International Conference on New Technologies, Mobility and Security.

[25] Torghabeh N.A., Totonchi M.R.A. Mobile base station management using fuzzy logic in wireless sensor networks. Proceedings of the International Conference on Computer Engineering and Technology.

[26] Kunarak S., Suleesathira R. Predictive RSS with fuzzy logic based vertical handoff algorithm in heterogeneous wireless networks. Proceedings of the International Symposium on Communications and Information Technologies.

[27] Fotouhi H., Alves M., Koubaa A., Baccour N. On a Reliable Handoff Procedure for Supporting Mobility in Wireless Sensor Networks. Proceedings of the 9th International Workshop on Real-Time Networks.

[28] Zinonos Z., Chrysostomou C., Vassiliou V. (2014). Wireless sensor networks mobility management using fuzzy logic. Ad Hoc Netw..

[29] Lee H.J., Wicke M., Kusy B., Gnawali O. (2015). Predictive Data Delivery to Mobile Users Through Mobility Learning in Wireless Sensor Networks. IEEE Trans. Veh. Technol..

[30] Al-Nidawi Y., Yahya H., Kemp A.H. (2016). Tackling Mobility in Low Latency Deterministic Multihop IEEE 802.15.4e Sensor Network. IEEE Sens. J..

[31] Yang D., Xu Y., Wang H., Zheng T., Zhang H., Zhang H., Gidlund M. (2015). Assignment of segmented slots enabling reliable real-time transmission in industrial wireless sensor networks. IEEE Trans. Ind. Electron..

[32] Fabbri F., Zuniga M., Puccinelli D., Marrón P. On the Optimal Blacklisting Threshold for Link Selection in Wireless Sensor Networks. Proceedings of the Wireless Sensor Networks—European Conference, Ewsn 2012.

[33] Lu J., Zhang G., Ruan D. (2007). Multi-Objective Group Decision Making: Methods, Software and Applications with Fuzzy Set Techniques.

[34] Tzeng G.H., Huang J.J. (2013). Fuzzy Multiple Objective Decision Making.

[35] Yager R.R. (1988). On ordered weighted averaging aggregation operators in multicriteria decisionmaking. IEEE Trans. Syst. Man Cybern..

[36] Youssef H., Sait S.M., Khan S.A. Fuzzy Evolutionary Hybrid Metaheuristic for Network Topology Design. Proceedings of the International Conference on Evolutionary Multi-Criterion Optimization.

[37] Bobillo F., Straccia U. (2013). Aggregation operators for fuzzy ontologies. Appl. Soft Comput..

[38] Mota V.F.S., Cunha F.D., Macedo D.F., Nogueira J.M.S., Loureiro A.A.F. (2014). Protocols, mobility models and tools in opportunistic networks: A survey. Comput. Commun..

